# Complete mitochondrial genome of the fungal pathogen *Fusarium oxysporum* f. sp. *palmarum* responsible for fusarium wilt of palms

**DOI:** 10.1128/mra.00070-25

**Published:** 2025-06-10

**Authors:** Marie-Gabrielle Ayika, Seemanti Chakrabarti, Braham Dhillon

**Affiliations:** 1 Department of Plant Pathology, University of Florida, Fort Lauderdale Research and Education Center 316814, Davie, Florida, USA; University of California Riverside, Riverside, California, USA

**Keywords:** Fusarium oxysporum, palm, pacbio, mitochondria

## Abstract

We present the complete mitochondrial genome of *Fusarium oxysporum* f. sp. *palmarum* (FOP), a fungal pathogen that causes fusarium wilt of palms. The 47.3 kb circular FOP mitogenome contains the 14 core genes typically conserved in fungal mitogenomes and two introns encoding for homing endonucleases.

## ANNOUNCEMENT

*Fusarium oxysporum* f. sp. *palmarum* (FOP) is a fungal pathogen responsible for fusarium wilt of palms, a lethal disease usually observed on queen palms (*Syagrus romanzoffiana*) and Mexican fan palms (*Washingtonia filifera*) (1). Symptoms of fusarium wilt manifest as one-sided leaf death that starts on older fronds and progresses upward in the canopy toward the spear leaf.

FOP, a member of the *F. oxysporum* species complex (FOSC), is morphologically indistinguishable from other FOSC members, complicating species-level identification (2). The advent of molecular tools has revolutionized fungal diagnostics, with DNA-based markers enabling accurate identification, early detection, and monitoring of pathogens in symptomatic and asymptomatic plant tissues (3, 4). Combining morphological methods with molecular tools has proven to be an effective strategy for diagnosing phytopathogens, including *Fusarium* species. The mitochondrial genome of FOP was sequenced and analyzed in order to explore its utility in designing a molecular diagnostics assay for this pathogen.

The FOP isolate 249A was originally collected in 2007 from a diseased Mexican fan palm growing at a field nursery in Lee County, Florida, and was deposited in the USDA-ARS Culture Collection (NRRL), accession NRRL 53543. Genomic DNA from this isolate was extracted from freeze-dried mycelium using a modified cetyltrimethylammonium bromide method (5). DNA was purified using the Qiagen G-Tip column purification kit (Catalog # 10223), followed by sequencing on the Pacific Biosciences Sequel IIe platform (PacBio, Menlo Park, CA, USA). A library was constructed with a barcoded adaptor using the SMRTbell Express Template Prep Kit 2.0 (PacBio) and sequenced on a single SMRTCell 8M. A total of 2,082 raw PacBio reads, totaling 11,287,452 base pairs and with an N50 read length of 5,295 bp, were generated. Reads of mitochondrial origin were identified using Tiara version 1.0.2 (6), followed by genome assembly, circularization, and rotation using MitoHifi version 3.2 (7) with *F. oxysporum* f. sp. *cubense* (GenBank accession LT571433) as a reference genome. Gene prediction and functional annotation were carried out using MFannot v.2 webserver (8) with genetic code 4 (Mold, Protozoan, and Coelenterate Mitochondrial; Mycoplasma/Spiroplasma) and visualized using OGDRAW v.1.3.1 (9).

**Fig 1:**
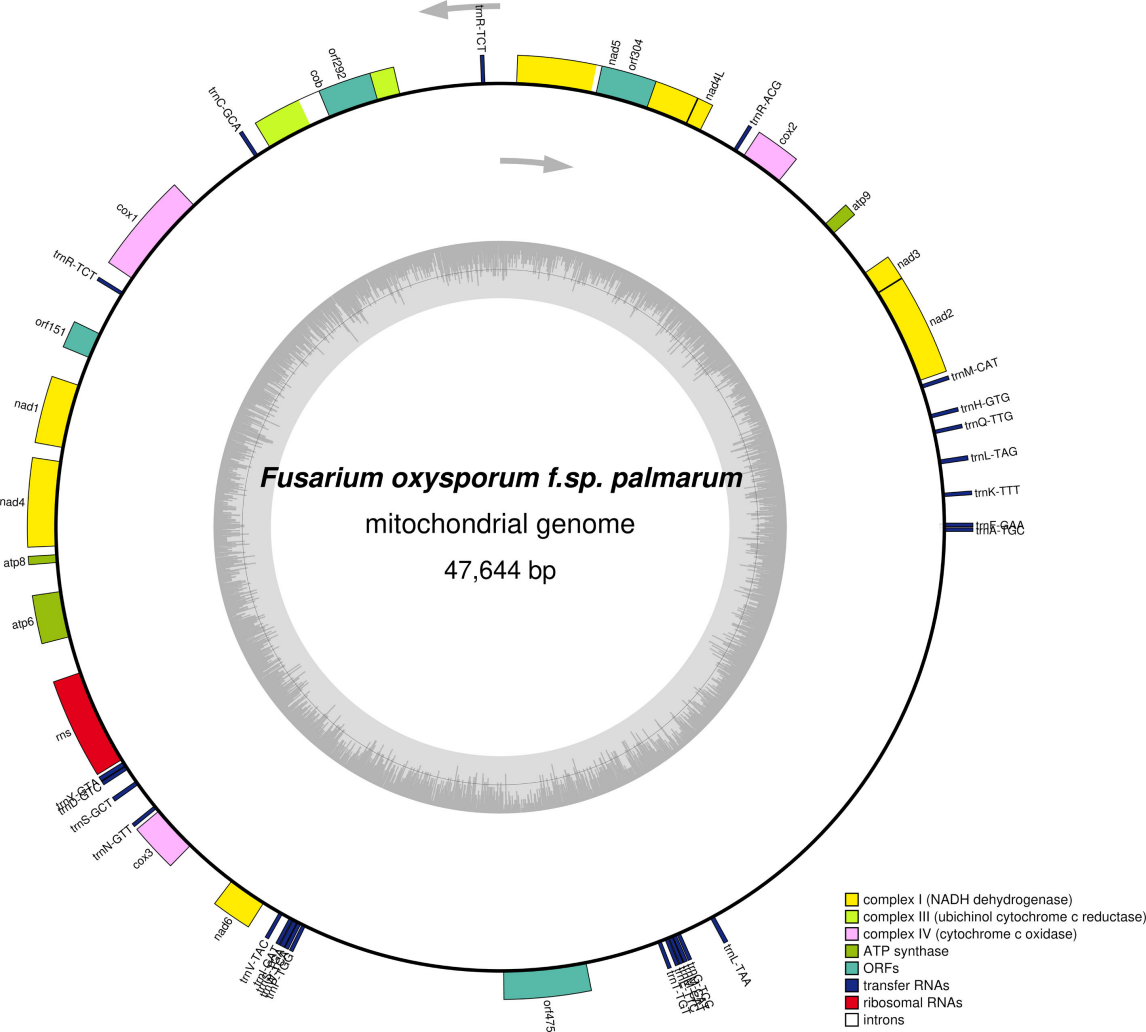
The mitochondrial genome of FOP. The inner circle shows the GC content, and all gene names have been italicized. The arrows represent the direction of transcription.

The mitogenome of FOP isolate 249A consists of a single circular chromosome of 47.3 kb with 239× coverage and a GC content of 32.3%. The *F. oxysporum* mitogenomes are typically compact, ranging between 45 and 52 kb, and are composed of highly structured genes (10). In total, 43 genes, including 14 core protein-coding genes, 1 rRNA, 23 tRNA, and 5 ORFs were predicted. The typical genes conserved in fungal mitogenomes*—atp6*, *atp8*, and *atp9* (ATP production); *nad1-6* and *nad4L* (oxidative phosphorylation); *cob* (apocytochrome b); and *cox1-3* (cytochrome C oxidase subunits)—were present in the FOP mitogenome (Fig. 1). Additionally, two introns encoding for homing endonucleases, GIY-YIG and LAGLIDADG, commonly found in fungal mitochondrial genomes, were identified in the *cob* and *nad5* genes, respectively.

## Data Availability

The mitochondrial genome sequence and annotation of *F. oxysporum* f. sp. *palmarum* isolate 249A have been deposited in GenBank under accession number PQ932601. The sequence raw reads were deposited in the NCBI Sequence Read Archive (SRA) under the BioProject accession number PRJNA1219565.
